# Specialized Pro-Resolving Mediators at the Ocular Surface: Biosynthesis, Mechanisms, and Therapeutic Potential

**DOI:** 10.3390/biology15141203

**Published:** 2026-07-21

**Authors:** Siddharth Gandhi, Arveen Shokravi, Sydney Buhrow, Michael Balas

**Affiliations:** 1 Faculty of Medicine, Queen’s University, Kingston, ON K7L 3L4, Canada; 2 Department of Medicine, University of Calgary, Calgary, AB T2N 2T8, Canada; 3 Department of Health Sciences, University of Western Ontario, London, ON N6A 3K7, Canada; 4 Department of Ophthalmology and Vision Sciences, University of Toronto, Toronto, M5T 3A9 ON, Canada

**Keywords:** specialized pro-resolving mediators, ocular surface, lipoxins, resolvins, maresins, dry eye disease, cornea, conjunctival goblet cells, lipidomics, omega-3 fatty acids

## Abstract

Specialized pro-resolving mediators (SPMs) are endogenous lipid mediators that actively orchestrate the resolution of inflammation rather than relying solely on broad suppression of inflammatory signaling. At the ocular surface, lipoxins, resolvins, protectins, maresins, and related pro-resolving mediators regulate epithelial repair, leukocyte responses, angiogenesis, conjunctival goblet-cell secretion, tear-film homeostasis, and corneal nerve regeneration. This review synthesizes evidence for local SPM biosynthesis and receptor signaling in the cornea and conjunctiva, together with tear lipid mediator profiling, with emphasis on anterior-segment and ocular-surface disease models. Particular attention is given to the endogenous lipoxin A4 circuit, resolvin-mediated control of corneal inflammation and viral keratitis, docosanoid regulation of corneal nerve regeneration, SPM modulation of conjunctival goblet-cell mucin secretion, tear lipid mediator signatures, and the translational development of resolvin E1 (RvE1)-based analogs for dry eye disease. Current evidence positions SPMs as mechanistically distinct therapeutic candidates for ocular-surface disease; however, clinical translation will require optimized delivery, biomarker-guided patient selection, and rigorous validation in disease-state cohorts.

## Simple Summary

Inflammation at the ocular surface contributes to dry eye disease and other disorders of the cornea and conjunctiva. The body resolves inflammation not only by reducing inflammatory signals but also by producing specialized pro-resolving mediators, which are molecules derived from fatty acids that actively promote tissue recovery. This review summarizes current evidence on these mediators at the ocular surface. Experimental studies suggest that lipoxins, resolvins, protectins, and maresins can support corneal wound healing, regulate immune-cell activity and abnormal blood-vessel growth, promote tear-film mucin secretion, and aid corneal nerve regeneration. Studies of human tears also suggest that these molecules may eventually serve as biomarkers, although the available evidence remains limited. Several resolvin-based treatments have progressed through preclinical or early clinical testing, but their effectiveness in patients is not yet fully established. Further research is needed to improve ocular delivery, identify patients most likely to benefit, and confirm safety and effectiveness in well-designed clinical studies. These mediators may ultimately complement existing anti-inflammatory treatments by helping restore normal ocular-surface function.

## 1. Introduction

Dry eye disease (DED) is one of several disorders affecting the ocular surface and has an estimated global prevalence of 5% to 50%, depending on the epidemiological criteria employed [1]. Although DED is a major focus of ocular-surface inflammatory research, chronic inflammation also contributes to other corneal and conjunctival disorders. Managing inflammation across these conditions remains a therapeutic challenge. Current anti-inflammatory treatments used for DED exhibit specific pharmacological limitations. Corticosteroids suppress inflammation but carry risks of intraocular pressure elevation and posterior subcapsular cataract formation with chronic use [2]. Cyclosporine A requires weeks to months to achieve clinical efficacy, and its therapeutic benefit varies among patient populations [3]. Newer agents such as lifitegrast target inflammatory pathways, including lymphocyte function-associated antigen 1 (LFA-1) and intercellular adhesion molecule 1 (ICAM-1) interactions, but act primarily by antagonizing inflammatory cell adhesion [4]. Consequently, there is an unmet need for therapies that promote the active resolution of inflammation.

The resolution of inflammation is an active, biosynthetically driven process governed by a superfamily of endogenous lipid molecules known as specialized pro-resolving mediators (SPMs) [5,6]. The SPM superfamily comprises several families enzymatically derived from polyunsaturated fatty acids. Lipoxins are generated from the omega-6 fatty acid arachidonic acid (AA). E-series resolvins are derived from the omega-3 fatty acid eicosapentaenoic acid (EPA). D-series resolvins, protectins, and maresins are synthesized from the omega-3 fatty acid docosahexaenoic acid (DHA) [5,6,7]. During an inflammatory response, lipid mediator class switching shifts biosynthesis away from predominantly pro-inflammatory prostaglandins and leukotrienes toward pro-resolving SPMs that limit leukocyte infiltration and restore tissue homeostasis [7].

The ocular surface is highly relevant to SPM biology. Mouse corneas generate endogenous lipoxin A4 (LXA4) and neuroprotectin D1/protectin D1 (NPD1/PD1). Following epithelial removal, corneal Alox15 messenger RNA (mRNA) expression (Alox15 encodes 12/15-lipoxygenase [12/15-LOX]), LXA4-receptor mRNA expression, and LXA4 formation are transiently abrogated and subsequently restored during wound healing [8]. These resident pro-resolving pathways have been proposed to help maintain the elevated anti-inflammatory tone of the immune-privileged cornea, amplify wound healing, regulate leukocyte responses, and support tissue homeostasis [4]. More broadly within the eye, DHA-enriched retinal photoreceptors provide abundant substrate for DHA-derived SPM pathways [4]. Building on prior reviews of ocular SPM biology [4], this review focuses on anterior-segment and ocular-surface studies, including SPM regulation of conjunctival goblet cell mucin secretion, corneal epithelial wound repair, corneal nerve regeneration, SPM signatures in human tears, and chemical and infectious keratitis models, with emphasis on translational potential.

## 2. Literature Search Strategy

This narrative review was informed by a structured search of PubMed from database inception through June 10, 2026. Search terms combined concepts related to specialized pro-resolving mediators, including “specialized pro-resolving mediator,” “lipoxin,” “resolvin,” “protectin,” “neuroprotectin,” “maresin,” “RX-10005,” and “RX-10045,” with ocular-surface terms including “ocular surface,” “cornea,” “conjunctiva,” “dry eye,” “tear,” “keratitis,” “goblet cell,” “corneal wound,” and “corneal nerve.” ClinicalTrials.gov was searched for completed or ongoing studies of ocular specialized pro-resolving mediator-based interventions. Reference lists of relevant reviews and included articles were examined, and forward citation searching was used to identify additional studies. Original in vitro, animal, human tear, clinical, and ocular drug-delivery studies were considered when directly relevant to specialized pro-resolving mediator biosynthesis, signaling, biological effects, or therapeutic translation at the ocular surface. Foundational nonocular studies were included selectively when required to describe established biosynthetic or receptor pathways. Evidence was synthesized narratively because of substantial heterogeneity in the mediators, experimental systems, disease models, and outcomes examined. No meta-analysis or formal risk-of-bias assessment was performed.

## 3. SPM Biosynthesis and Receptors: Overview

### 3.1. Biosynthetic Pathways

**Figure 1:**
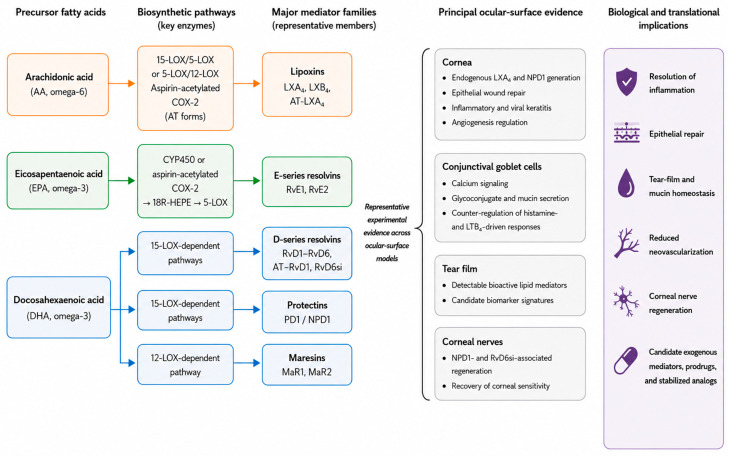
Conceptual overview of specialized pro-resolving mediator biosynthesis and ocular-surface effects. Arachidonic acid, eicosapentaenoic acid, and docosahexaenoic acid serve as precursors for lipoxins, E-series resolvins, D-series resolvins, protectins, and maresins through the key enzymatic pathways shown. Experimental evidence from corneal, conjunctival goblet-cell, tear-film, and corneal nerve models links these mediator families to endogenous lipid-mediator generation, epithelial wound repair, regulation of inflammatory and viral keratitis, angiogenesis, glycoconjugate and mucin secretion, tear lipid-mediator signatures, and corneal nerve regeneration. The depicted associations are representative rather than exclusive, and not every mediator family has been evaluated in every ocular-surface compartment or experimental context. Collectively, these pathways provide a biological rationale for investigating exogenous mediators, prodrugs, and stabilized analogs as potential ocular-surface therapies. Abbreviations: AA, arachidonic acid; AT, aspirin-triggered; COX-2, cyclooxygenase-2; CYP450, cytochrome P450; DHA, docosahexaenoic acid; EPA, eicosapentaenoic acid; 18R-HEPE, 18R-hydroxyeicosapentaenoic acid; LOX, lipoxygenase; LTB4, leukotriene B4; LXA4, lipoxin A4; LXB4, lipoxin B4; MaR1, maresin 1; MaR2, maresin 2; NPD1, neuroprotectin D1; PD1, protectin D1; RvD1–RvD6, D-series resolvins 1–6; RvD6si, resolvin D6 stereoisomer; RvE1 and RvE2, E-series resolvins 1 and 2.

The biosynthesis of SPMs involves coordinated enzymatic conversion of polyunsaturated fatty acids. Lipoxins are generated from AA through transcellular lipoxygenase pathways, including interactions between 15-lipoxygenase (15-LOX) and 5-lipoxygenase (5-LOX), or between 5-LOX and 12-lipoxygenase (12-LOX), yielding LXA4 and lipoxin B4 (LXB4) [6,7]. Aspirin-acetylated cyclooxygenase-2 (COX-2) can initiate the formation of aspirin-triggered lipoxins by generating 15R-hydroxyeicosatetraenoic acid (15R-HETE), which is subsequently converted to 15-epi-lipoxins such as 15-epi-LXA4; these epimers resist metabolic inactivation and signal through the lipoxin A4 receptor/formyl peptide receptor 2 (ALX/FPR2) [6,7]. EPA is converted via cytochrome P450 (CYP450) or aspirin-acetylated COX-2 pathways to 18R-hydroxyeicosapentaenoic acid (18R-HEPE), which is further transformed by 5-LOX into E-series resolvins, including resolvin E1 (RvE1) and resolvin E2 (RvE2) [7]. DHA is converted through 15-LOX-dependent pathways to 17S-hydroperoxydocosahexaenoic acid/17S-hydroxydocosahexaenoic acid intermediates that give rise to D-series resolvins, resolvin D1 (RvD1) through resolvin D6 (RvD6) [7]. Aspirin-triggered D-series resolvins, including aspirin-triggered resolvin D1 (AT-RvD1), are generated through analogous pathways involving 17R-configured intermediates [7]. DHA is also metabolized through 15-LOX-dependent epoxidation pathways to form protectins, most notably PD1/NPD1 [7]. Maresins, including maresin 1 (MaR1) and maresin 2 (MaR2), are DHA-derived mediators first identified in macrophage-associated resolution pathways; their biosynthesis is initiated by 12-LOX conversion of DHA to 14-hydroperoxy-DHA, followed by formation of a 13S,14S-epoxy-maresin intermediate that is enzymatically converted to MaR1 or MaR2 [9,10]. Together, these pathways establish the biochemical framework for the receptor-specific and tissue-level effects discussed below. Figure 1 provides an integrated conceptual overview linking precursor fatty acids and major SPM families with ocular-surface target compartments and downstream biological and translational implications.

### 3.2. Receptors at the Ocular Surface

The biological actions of SPMs are mediated largely through G-protein-coupled receptors (GPCRs) [5,6]. At the ocular surface, ALX/FPR2 is one of the best-characterized SPM receptors, mediating LXA4 signaling and participating in RvD1, AT-RvD1, and annexin A1 signaling in conjunctival goblet-cell systems [11,12,13]. Mouse corneas express Alox15 and ALX/FPR2 mRNA, and epithelial injury dynamically alters expression of these pathways during wound repair [8]. Corneal FPR2 expression has also been assessed in murine lipopolysaccharide (LPS)-induced keratitis models [14]. In conjunctival goblet-cell systems, RvE1 signals through chemerin chemokine-like receptor 1 (CMKLR1), also known as chemerin receptor 23/E-series resolvin receptor 1 (ChemR23/ERV1), and interacts with leukotriene B4 receptor 1 (BLT1) to counter-regulate leukotriene B4-dependent calcium and secretory responses [15,16]. RvD1 receptor use is species dependent: human conjunctival goblet-cell studies identify G-protein-coupled receptor 32 (GPR32) as a principal RvD1 receptor, whereas rat studies show that RvD1 and AT-RvD1 signal through ALX/FPR2; additional work suggests that ALX/FPR2 may also participate in human RvD1 signaling [11,13,15]. Maresin receptor biology at the ocular surface remains incompletely defined [17,18]. In rat conjunctival goblet cells, MaR2 requires BLT1 for calcium signaling, whereas MaR1 uses both BLT1 and ALX/FPR2 for calcium signaling and BLT1 for glycoconjugate secretion [17,18]. Collectively, expression of SPM biosynthetic enzymes and receptors in corneal and conjunctival tissues supports the existence of local pro-resolving circuits within the ocular surface [4,8]. The major SPM families, precursor pathways, representative receptors, and supporting ocular-surface evidence are summarized in Table 1.

## 4. SPMs in the Cornea

The cornea provides the most extensively characterized ocular-surface model of SPM biology. Current evidence spans endogenous lipoxin circuits, epithelial repair, inflammatory and infectious keratitis, pathological angiogenesis, and corneal nerve regeneration.

### 4.1. The Endogenous Lipoxin A4 Circuit

The cornea possesses a local endogenous pro-resolving biosynthetic circuit. Gronert and colleagues established that mouse corneas generate endogenous LXA4 and NPD1 and that epithelial removal abrogates corneal Alox15 and LXA4-receptor mRNA expression, together with LXA4 formation; these are subsequently restored during wound healing [8]. In LPS-challenged corneal wounds, Alox15−/− mice exhibit exacerbated inflammation and impaired epithelial wound healing; topical LXA4 partially restores wound healing [26]. The actions of LXA4 are linked to the cytoprotective heme-oxygenase system: 12/15-LOX deletion impairs heme oxygenase-1 induction, LXA4 restores heme oxygenase-1 expression, and heme oxygenase-2 deficiency reduces endogenous LXA4 formation, indicating interdependence between these counter-regulatory systems during corneal wound healing [26].

Beyond basal homeostasis, LXA4 serves as a downstream effector for classic growth-factor signaling. Epidermal growth factor (EGF)-stimulated epithelial wound healing is partially mediated through a 12/15-LOX-LXA4 pathway, and LXA4-dependent wound closure requires extracellular signal-regulated kinase 1/2 (ERK1/2) and p38 mitogen-activated protein kinase signaling [27]. A functional endogenous LXA4 circuit also constrains pathological angiogenesis during chronic injury, with genetic deletion of 15-LOX or 5-LOX exacerbating inflammatory neovascularization and topical LXA4 reducing vascular endothelial growth factor A (VEGF-A), fms-related receptor tyrosine kinase 4 (FLT4), and inflammatory angiogenesis [28].

Topical LXA4 administration in a rat model of severe alkali burn reduced corneal opacity, neovascularization, and hyphema [29]. Ribonucleic acid (RNA) sequencing and protein analyses showed that LXA4 reduced interleukin (IL)-1β, IL-6, matrix metalloproteinase 9 (MMP-9), and VEGF-A, while upregulating repair-associated pathways, including keratinization and epidermal growth factor receptor-family signaling [29]. Separately, flow cytometry showed that LXA4 increased the proportion of type 2 macrophages (M2) relative to type 1 macrophages (M1) in blood-isolated monocytes after alkali injury [29]. These findings indicate that the endogenous LXA4 pathway can be targeted experimentally to promote corneal healing after chemical injury [29].

### 4.2. Resolvins in Corneal Inflammation and Wound Healing

Resolvins exhibit anti-inflammatory activity in corneal epithelial cells [3]. In cultured human corneal epithelial cells, RvD1 reduced polyinosinic:polycytidylic acid-induced messenger RNA expression of tumor necrosis factor alpha (TNF-α), IL-6, IL-1β, and IL-8 [3]. At the protein level, RvD1 significantly reduced TNF-α, IL-6, and IL-8 secretion, whereas the reduction in IL-1β protein did not reach statistical significance at the reported concentration [3]. Significant dose–response effects were reported for TNF-α and IL-1β [3]. The authors associated these anti-inflammatory effects with regulation of nuclear factor kappa B (NF-κB) signaling, based on changes in inhibitory kappa B alpha (IκBα) expression [3].

In vivo studies support the anti-inflammatory potential of RvD1 in ocular inflammatory models [14]. In a mouse model of LPS-induced keratitis, intrastromal RvD1 reduced clinical keratitis scores in a dose-dependent manner [14]. RvD1 also reduced polymorphonuclear leukocyte accumulation, myeloperoxidase activity, and local expression of C-X-C motif chemokine ligand 1 (CXCL1), also termed keratinocyte-derived chemokine, while preserving IL-10 levels [14]. In addition, RvD1 restored connexin-43 expression in the corneal epithelium and shifted macrophage-associated markers from an M1-like toward an M2-like profile [14]. Corneal formyl peptide receptor 2 (FPR2) expression was assessed in this model, and the authors interpreted the protective effect as consistent with FPR2-associated signaling and macrophage-leukocyte regulatory activity [14].

Resolvins also show regenerative activity in metabolically compromised corneal tissue [22]. In a streptozotocin-induced type 1 diabetic mouse model, topical RvD1 accelerated corneal epithelial wound closure and restored corneal mechanical sensitivity [22]. The FPR2 antagonist WRW4 reversed RvD1-associated improvements in epithelial regeneration and antioxidant responses, supporting an FPR2-dependent component of the effect [22]. RvD1 also reduced inflammatory-cell infiltration, myeloperoxidase activity, TNF-α and IL-1β expression, and oxidative-stress markers while increasing the proportion of M2 macrophages in injured diabetic corneas [22]. In parallel, RvD1 restored phosphorylation of epidermal growth factor receptor (EGFR), sirtuin 1 (Sirt1), and Ki67 expression, and reduced reactive oxygen species accumulation and nicotinamide adenine dinucleotide phosphate (NADPH) oxidase 2/4 expression [22]. Diabetic keratopathy is characterized by delayed epithelial repair and impaired corneal sensation; these preclinical findings position RvD1 as a therapeutic candidate for further study [22].

### 4.3. SPMs in Herpes Simplex Keratitis

Stromal keratitis caused by herpes simplex virus 1 (HSV-1) is an immunopathological condition in which neutrophils and cluster of differentiation 4-positive (CD4+) T cells contribute substantially to corneal inflammation and lesion development [30,31]. In murine HSV-1 keratitis models, exogenous SPMs reduced inflammatory lesion severity [30,31]. Topical administration of the methyl ester prodrug of RvE1, RX-10005, reduced the severity of viral stromal keratitis and corneal neovascularization in mice [30]. RvE1 treatment also decreased the influx of neutrophils and pathogenic CD4+ T cells and increased corneal production of the anti-inflammatory cytokine IL-10 [30].

AT-RvD1 produced additional regulatory effects in HSV-1-induced stromal keratitis [31]. Topical AT-RvD1 reduced corneal expression of pro-inflammatory cytokines and chemokines, including IL-1β, IL-6, IL-12, CXCL1, monocyte chemoattractant protein 1 (MCP-1), and macrophage inflammatory protein 2 (MIP-2), as well as the angiogenesis-associated mediators VEGF-A and MMP-9 [31]. AT-RvD1 also attenuated phosphorylated signal transducer and activator of transcription 1 (pSTAT1) signaling and reduced T helper type 1 (Th1)- and T helper type 17 (Th17)-associated corneal inflammatory responses [31]. In addition, AT-RvD1 downregulated pro-inflammatory microRNAs (miRNAs), including miR-155, miR-132, and miR-223, in infected corneas [31]. These findings provide ocular evidence that SPM therapy can modulate inflammatory miRNA networks during viral keratitis [31].

### 4.4. NPD1 and RvD6si in Corneal Nerve Regeneration

The regeneration of corneal nerves after surgical or pathological injury requires neurotrophic coordination. DHA serves as a precursor in this environment. When applied in conjunction with pigment epithelium-derived factor (PEDF), DHA stimulates the synthesis of NPD1 in the cornea [24,25]. Localized NPD1 production enhances nerve regeneration by increasing expression of brain-derived neurotrophic factor (BDNF), nerve growth factor (NGF), and semaphorin 7A [25].

Exploration of DHA metabolism in injured mouse corneas identified a stereoisomer of resolvin D6, designated RvD6si in the original primary study and subsequently referred to as RvD6i in a later review [23,25]. To maintain consistent and specific nomenclature, RvD6si is used throughout this review. Topical RvD6si accelerated corneal epithelial wound closure, enhanced corneal nerve regeneration, and improved recovery of corneal mechanical sensitivity in mouse injury models [23]. RNA sequencing of trigeminal ganglia after topical RvD6si treatment showed reduced expression of pain-associated genes, including Calcb and Tac1, together with increased expression of genes linked to axon growth, neurogenesis, or sensory function, including C9orf72, Gpm6A, Rictor, and Trpm8 [23]. These findings suggest that RvD6si modulates cornea-trigeminal ganglion signaling in a manner consistent with improved sensory recovery and reduced pain-related molecular signaling [23,25]. Because corneal nerve damage after refractive surgery, diabetes, infection, and other insults can impair epithelial wound healing, reduce tear secretion, and contribute to neuropathic ocular pain, RvD6si and related DHA-derived mediators are promising preclinical candidates for restoring corneal sensory homeostasis [25].

## 5. SPMs in the Conjunctiva and Tear Film

Complementing the corneal literature, studies of the conjunctiva and tear film indicate that pro-resolving mediators also regulate secretory homeostasis and may provide measurable biomarkers of ocular-surface inflammatory activity.

### 5.1. SPM Regulation of Goblet Cell Mucin Secretion

Between 2013 and 2022, multiple studies identified cultured conjunctival goblet cells as direct targets of pro-resolving mediator signaling [11,12,13,15,16,17,18,19]. Collectively, these studies show that the SPM families tested to date, including D-series resolvins, E-series resolvins, lipoxins, and maresins, as well as the pro-resolving protein mediator annexin A1, regulate goblet-cell calcium signaling and high-molecular-weight glycoconjugate secretion [11,12,13,15,16,17,18,19]. These findings are consistent with a role for pro-resolving mediators in maintaining tear-film mucin homeostasis under physiological conditions and limiting dysregulated secretory responses during inflammation [11,12,13,15,16,17,18,19]. The comparative effects and signaling pathways of these mediators in conjunctival goblet cells are summarized in Table 2.

RvD1 increases intracellular calcium concentration ([Ca^2+^]_i_) and stimulates secretion of high-molecular-weight glycoconjugates, including mucins, in cultured rat and human conjunctival goblet cells [11,15]. Pharmacologic inhibition of EGFR reduced RvD1-induced [Ca^2+^]_i_ elevation in both rat and human cells; in rat goblet cells, EGFR inhibition also blocked RvD1-stimulated glycoconjugate secretion, protein kinase B (AKT) activation, and ERK1/2 activation [15]. RvD1 and AT-RvD1 also inhibited histamine-stimulated [Ca^2+^]_i_ elevation and glycoconjugate secretion in cultured rat and human goblet cells [11]. In rat goblet cells, both mediators additionally inhibited histamine-induced ERK1/2 activation [11].

The E-series resolvin RvE1 similarly stimulates glycoconjugate secretion and [Ca^2+^]_i_ signaling in cultured rat conjunctival goblet cells through phospholipase C (PLC), phospholipase D (PLD), and phospholipase A2 (PLA2) pathways [19]. Although EGFR inhibition reduced RvE1-induced ERK1/2 activation in rat goblet cells, RvE1 did not transactivate EGFR to drive its [Ca^2+^]_i_ or glycoconjugate-secretory responses, distinguishing its signaling profile from that of RvD1 [15]. In an inflammatory setting, RvE1 counter-regulated leukotriene B4 (LTB4)-induced [Ca^2+^]_i_ elevation and high-molecular-weight glycoprotein secretion in rat conjunctival goblet cells [16].

LXA4 stimulates [Ca^2+^]_i_ mobilization, glycoconjugate secretion, and ERK1/2 activation in cultured human conjunctival goblet cells through LXA4 receptor/formyl peptide receptor 2 (ALX/FPR2) [13]. LXA4 also reduced histamine-stimulated [Ca^2+^]_i_ elevation and glycoconjugate secretion in rat and human goblet cells and inhibited histamine-stimulated ERK1/2 activation in rat goblet cells [13]. This counter-regulatory effect required β-adrenergic receptor kinase 1 (βARK1)-dependent modulation of histamine H1-receptor signaling [13].

Subsequent studies extended this pro-resolving goblet-cell paradigm to the maresin family. MaR1 elevates [Ca^2+^]_i_ and stimulates glycoconjugate secretion in rat conjunctival goblet cells through signaling involving PLC, inositol trisphosphate (IP3), protein kinase C (PKC), PLD, calcium/calmodulin-dependent protein kinase II (CaMKII), and ERK1/2 [17,18]. MaR2 also stimulates [Ca^2+^]_i_ mobilization and secretion but uses a distinct, partially overlapping signaling profile [18]. In rat conjunctival goblet cells, MaR2 uses BLT1-dependent cyclic adenosine monophosphate/protein kinase A (cAMP/PKA), PLD, PLC-PKC, and PLA2 pathways to increase [Ca^2+^]_i_; PLC-IP3 signaling is not required for its calcium response but contributes to MaR2-stimulated secretion [18]. Both MaR1 and MaR2 counter-regulate histamine-driven goblet-cell responses, whereas MaR1, but not MaR2, reduces LTB4-stimulated [Ca^2+^]_i_ elevation [17,18].

Finally, annexin A1, a pro-resolving protein mediator that shares ALX/FPR2-dependent signaling with several lipid SPMs, increases [Ca^2+^]_i_ and glycoconjugate secretion in cultured rat conjunctival goblet cells [12]. Its Ca^2+^ and secretory effects are inhibited by ALX/FPR2 blockade and depend on PLC-linked signaling, while ERK1/2, PLA2, and PLD contribute specifically to annexin A1-stimulated secretion [12].

Across the pro-resolving mediators tested in conjunctival goblet-cell models, a common pattern emerges: they stimulate basal glycoconjugate secretion while counter-regulating selected inflammatory secretory responses induced by histamine or LTB4 [11,12,13,15,16,17,18,19]. The exact inflammatory stimulus and signaling pathway targeted differ among mediators, but the collective findings support a role in preserving mucin-layer homeostasis [11,12,13,15,16,17,18,19]. These mechanistic data provide a rationale for further testing of SPM-based therapies in allergic conjunctivitis and other ocular-surface diseases characterized by dysregulated mucin secretion [11,12,13,15,16,17,18,19].

### 5.2. SPM Signatures in Human Tears

English and colleagues performed targeted liquid chromatography-tandem mass spectrometry (LC-MS/MS) metabololipidomic profiling of human emotional tears from 12 healthy individuals [32]. Their analysis identified pro-inflammatory lipid mediators, including prostaglandins and LTB4, together with pro-resolving mediators, including D-series resolvins, protectin D1/neuroprotectin D1 (PD1/NPD1), and LXA4, at concentrations reported to be bioactive [32].

MaR1 and MaR2 were not detected in these emotional tear samples despite their demonstrated bioactivity in conjunctival goblet-cell models [17,18,32]. The study did not determine whether this non-detection reflects local tissue-restricted maresin activity, concentrations below the analytical detection threshold in tears, variation with inflammatory state or diet, or a limited contribution of maresins to emotional tear fluid under basal conditions [32].

The metabololipidomic analysis also identified sex-associated differences in tear lipid mediator composition [32]. Principal component analysis associated RvD1, RvD2, RvD5, and PD1/NPD1 with male donor samples, whereas LXA4 and aspirin-triggered LXA4 were associated with female donor samples [32]. These multivariate associations should not be interpreted as indicating higher absolute concentrations in female tears. Across the measured SPMs, concentrations were lower overall in female than male donors, yielding a lower total SPM:LTB4 ratio in female samples [32]. Because this exploratory study included only 12 healthy donors and analyzed emotional tears, these observations should be regarded as hypothesis-generating and should not be extrapolated to sex differences in DED prevalence or ocular-surface disease susceptibility [1,32]. Characterizing tear SPM profiles in larger, well-phenotyped cohorts with DED, allergic conjunctivitis, ocular graft-versus-host disease, Sjögren-related dry eye, or post-surgical inflammation will be important for determining whether lipid mediator profiling has diagnostic, prognostic, or therapeutic-stratification value [1,32].

## 6. Translational Progress and Therapeutic Potential

Together, the corneal, conjunctival, and tear-film findings provide a rationale for translating SPM biology into ocular-surface therapeutics. RvE1, delivered topically as its methyl ester prodrug RX-10005, demonstrated efficacy in murine models of dry eye disease. In a desiccating-stress model, RX-10005 reduced the desiccation-induced increase in corneal Oregon Green Dextran permeability/staining by 80% compared with untreated disease controls and prevented the loss of conjunctival goblet cells [20]. In a separate desiccating-stress dry eye model, topical RvE1 improved tear production, increased superficial corneal epithelial-cell density, and reduced COX-2 expression and corneal infiltration by CD4+ T cells and cluster of differentiation 11b-positive (CD11b-positive) myeloid cells [21]. The RvE1 analog RX-10045 was also evaluated in a rabbit photorefractive keratectomy (PRK) haze model. In a masked study, topical 0.1% RX-10045 administered immediately after −9-diopter PRK and every four hours for five days reduced corneal haze; however, its effect on myofibroblast generation was partial, with apparent inhibition of bone-marrow-derived, but not keratocyte-derived, myofibroblast precursors [33,34].

RX-10045 advanced to human clinical testing for dry eye disease. Clinical trial registry records identify two completed Phase II studies: a 232-patient randomized, placebo-controlled study conducted under everyday environmental conditions and controlled adverse environment provocation, and a later randomized Phase II study of 0.09% RX-10045 ophthalmic solution [35,36]. Company-reported results for the earlier study described RX-10045 as safe and well tolerated, with dose-dependent improvements in selected signs and symptoms [37]. However, detailed peer-reviewed efficacy reports for the completed dry eye trials were not identified in the literature reviewed [38]. Accordingly, the clinical efficacy of RX-10045 should be considered incompletely characterized. Nevertheless, RX-10045 advanced to Phase II clinical testing for dry eye disease [38].

The available evidence suggests two complementary therapeutic strategies: enhancing endogenous pro-resolving pathways and administering exogenous mediators or metabolically stabilized analogs. Proof of concept for endogenous pathway enhancement has been demonstrated in preclinical corneal models. Epidermal growth factor stimulates 12/15-LOX-dependent LXA4 synthesis during epithelial repair, while pigment epithelium-derived factor combined with DHA promotes NPD1 formation and corneal nerve regeneration [24,25,27]. Aspirin-acetylated COX-2 can also redirect lipid-mediator biosynthesis toward aspirin-triggered lipoxins and resolvins [6,7]. However, it has not yet been established that endogenous ocular-surface SPM production can be selectively and safely augmented in patients. Increasing precursor availability alone may also be insufficient because mediator generation depends on local enzyme expression, cellular interactions, and inflammatory context.

A parallel strategy is the direct administration of exogenous SPMs or more stable derivatives. Topical LXA4, RvD1, RvE1, and aspirin-triggered RvD1 have demonstrated activity in preclinical models of corneal injury, dry eye disease, inflammatory and viral keratitis, and impaired nerve repair [20,21,22,23,24,25,26,27,28,29,30,31]. Separately, NPD1 and RvD6si have demonstrated corneal nerve-regenerative effects in preclinical models [23,24,25]. The methyl ester prodrug RX-10005 and the synthetic RvE1 analog RX-10045 further illustrate efforts to improve ocular penetration, metabolic stability, and clinical feasibility [20,34,35,36,37,38,39,40]. Collectively, these findings support both endogenous pathway amplification and direct mediator replacement as potential therapeutic approaches. Future studies should determine whether one strategy is superior or whether combining pathway enhancement with exogenous analog delivery produces more durable resolution of ocular-surface inflammation.

Drug delivery remains an important translational issue. The corneal epithelium limits topical drug penetration, which motivated development of RX-10005 as a methyl ester prodrug intended to enhance ocular-surface uptake [20]. In transporter studies, RX-10045 behaved as a substrate/inhibitor of multidrug resistance-associated protein 2 (MRP2) and breast cancer resistance protein (BCRP), and it interacted with organic cation transporter 1 (OCT1) in human corneal epithelial cells [39]. Whether these transporter interactions materially limit ocular exposure or clinical efficacy in dry eye disease remains unproven [39]. An aqueous micellar RX-10045 formulation was developed to improve solubilization and topical tissue distribution and was reported to be well tolerated in rabbit ocular studies [40]. Nanoparticles, nanomicelles, and liposomes are reasonable formulation strategies for overcoming ocular delivery barriers, but they require direct evaluation with SPM analogs [39,40]. Patient selection is another unresolved issue: broad dry-eye inclusion criteria may obscure therapeutic effects in subgroups with impaired resolution biology. A precision-medicine approach based on tear lipid mediator profiles remains hypothetical and has not yet been established in clinical trials.

SPM-based therapeutics are conceptually distinct from conventional anti-inflammatory drugs. Rather than broadly suppressing inflammatory signaling, they are intended to engage endogenous resolution pathways that limit leukocyte recruitment, promote epithelial repair, and support tissue homeostasis [5,6,7]. This mechanism could be advantageous in ocular-surface disease, where therapies that reduce inflammation while preserving or restoring barrier function are needed. However, claims of faster onset, steroid-sparing efficacy, or reduced long-term toxicity require additional controlled clinical trials with validated signs, symptoms, safety endpoints, and biomarker-defined patient subgroups.

## 7. Future Directions and Knowledge Gaps

Despite substantial preclinical progress, several mechanistic and translational gaps remain in ocular-surface SPM biology [4]. Targeted LC-MS/MS profiling of SPMs in human tears remains limited, with the key direct study analyzing emotional tears from 12 healthy donors [32]. Future studies should profile tears from patients with DED, allergic conjunctivitis, post-surgical inflammation, ocular graft-versus-host disease, and Sjögren syndrome-associated dry eye to determine whether lipid mediator signatures have diagnostic, disease-stratification, prognostic, or therapeutic-response value.

The sex-associated tear lipid mediator signatures described above should be validated in larger, well-phenotyped cohorts, because the initial metabololipidomic study included only 12 healthy donors and analyzed emotional tears [32]. It remains unknown whether these signatures reflect hormonal regulation, differential lipoxygenase expression, substrate availability, tear-collection conditions, analytical detection limits, or other metabolic factors. Until replicated in larger disease-state cohorts, these signatures should not be interpreted as explaining sex differences in DED prevalence or ocular-surface disease susceptibility. If validated, sex-associated tear lipid mediator profiles could generate mechanistic hypotheses regarding differential susceptibility to ocular-surface inflammation. Future clinical trials should consider whether tear biomarker profiles can identify patient subgroups more likely to benefit from pro-resolving therapies.

Optimizing drug delivery is another major research need. Future formulation studies should test whether lipid nanoparticles, nanoemulsions, nanomicelles, or sustained-release mucoadhesive systems improve ocular residence time, epithelial penetration, chemical stability, and tissue exposure of topically applied SPM analogs [39,40]. In parallel, the observation that AT-RvD1 reduced corneal expression of inflammatory miRNAs, including miR-155, miR-132, and miR-223, during herpes simplex virus 1 keratitis identifies a potentially important mechanistic area for investigation [31]. Whether SPM-driven miRNA modulation is causal in lesion resolution, useful as a biomarker, or therapeutically targetable remains unknown.

Ocular-surface SPM circuits have not been systematically characterized in several chronic and cicatrizing conditions, including contact lens-related keratopathy, Stevens-Johnson syndrome/toxic epidermal necrolysis, and limbal stem cell deficiency [4]. Finally, single-cell RNA sequencing and spatial transcriptomics could identify corneal and conjunctival cell populations that express SPM biosynthetic enzymes and receptors across anatomically distinct ocular-surface microenvironments, including limbal stem-cell niches, the central corneal epithelium, and discrete bulbar and palpebral conjunctival epithelial and goblet-cell regions. Spatially resolved comparisons of these compartments, coupled with complementary spatial lipidomics or cell-resolved LC-MS/MS approaches, could determine whether mediator production and receptor expression are enriched within specific corneal, limbal, or conjunctival niches. Addressing these gaps could provide a molecular framework for developing targeted, biomarker-informed pro-resolving therapeutic strategies.

## 8. Conclusions

The characterization of specialized pro-resolving mediators represents a conceptual shift from suppressing inflammation toward actively engaging endogenous resolution pathways. The cornea and ocular surface possess local pro-resolving biosynthetic circuits that generate protective lipid mediators, contributing to immune regulation, epithelial repair, and tissue homeostasis. Recent studies have expanded this framework by demonstrating SPM regulation of conjunctival goblet cell glycoconjugate secretion, DHA-derived docosanoid promotion of corneal nerve regeneration, attenuation of viral keratitis accompanied by modulation of inflammatory miRNA networks, and preliminary sex-associated differences in human tear lipid mediator signatures. Successful clinical translation will require optimized ocular delivery systems, refined patient selection guided by tear-film biomarkers, and careful testing of rational combination strategies. Therapeutics based on pro-resolving lipid mediators hold promise as complements to current anti-inflammatory approaches for ocular surface disease, particularly in settings where inflammation, epithelial injury, mucin dysregulation, and impaired tissue repair coexist.

## Figures and Tables

**Table 1:** SPM Families Relevant to the Ocular Surface.

SPM Family	Representative Members	Precursor Fatty Acid	Key Biosynthetic Pathway	Receptor(s)	Ocular-Surface Evidence
Lipoxins	LXA4, LXB4, AT-LXA4/15-epi-LXA4	AA, omega-6	15-LOX/5-LOX or 5-LOX/12-LOX; aspirin-acetylated COX-2 → 15R-HETE → AT forms [6,7]	ALX/FPR2 [6,7]	Mouse corneas generate endogenous LXA4 and express Alox15 and ALX/FPR2 transcripts during injury and repair; human conjunctival goblet cells respond to LXA4 through ALX/FPR2 [8,13]
E-series resolvins	RvE1, RvE2	EPA, omega-3	CYP450 or aspirin-acetylated COX-2 → 18R-HEPE; 5-LOX [7]	ChemR23/ERV1 (CMKLR1), BLT1 [5,7]	Functional evidence in conjunctival goblet cells and dry-eye models; RvE1 signaling characterized in goblet cells, and RvE1 improves barrier and inflammatory outcomes in desiccating-stress models [15,16,19,20,21]
D-series resolvins	RvD1-RvD6, AT-RvD1, RvD6si *	DHA, omega-3	15-LOX-derived 17S-HpDHA/17S-HDHA; 5-LOX; aspirin-triggered 17R epimers [7]	RvD1: GPR32 in human goblet cells; ALX/FPR2 in rat goblet cells. Other D-series receptors vary or remain less defined [11,15]	RvD1 has corneal anti-inflammatory/wound-healing and goblet-cell evidence; RvD6si is implicated in corneal nerve regeneration [3,11,14,15,22,23]
Protectins	PD1/NPD1	DHA, omega-3	15-LOX-dependent 17S-HpDHA epoxidation pathway [7]	No ocular-surface receptor established	Mouse corneas generate NPD1; NPD1/PEDF plus DHA are linked to epithelial repair and corneal nerve-regeneration pathways [8,24,25]
Maresins	MaR1, MaR2	DHA, omega-3	12-LOX → 14S-HpDHA → 13S,14S-epoxy-maresin intermediate → MaR1 or MaR2 [9,10]	MaR1: BLT1 and ALX/FPR2 for calcium signaling; BLT1 for secretion. MaR2: BLT1 for calcium signaling; secretion receptor unresolved [17,18]	MaR1 and MaR2 regulate calcium signaling and glycoconjugate secretion in rat conjunctival goblet cells [17,18]

Abbreviations: AA, arachidonic acid; ALX/FPR2, lipoxin A4 receptor/formyl peptide receptor 2; AT, aspirin-triggered; BLT1, leukotriene B4 receptor 1; ChemR23/ERV1 (CMKLR1), chemerin chemokine-like receptor 1/E-series resolvin receptor 1 (chemokine-like receptor 1); COX-2, cyclooxygenase-2; CYP450, cytochrome P450; DHA, docosahexaenoic acid; EPA, eicosapentaenoic acid; GPR32, G-protein-coupled receptor 32; HDHA, hydroxydocosahexaenoic acid; HEPE, hydroxyeicosapentaenoic acid; HpDHA, hydroperoxydocosahexaenoic acid; LOX, lipoxygenase; LXA4, lipoxin A4; LXB4, lipoxin B4; MaR1, maresin 1; MaR2, maresin 2; NPD1, neuroprotectin D1; PD1, protectin D1; RvD1-RvD6, D-series resolvins 1–6; RvD6si, resolvin D6 stereoisomer; RvE1/RvE2, E-series resolvins 1 and 2; SPM, specialized pro-resolving mediator; 15R-HETE, 15R-hydroxyeicosatetraenoic acid; 17S-HDHA, 17S-hydroxydocosahexaenoic acid; 17S-HpDHA, 17S-hydroperoxydocosahexaenoic acid; 5-LOX, 5-lipoxygenase; 12-LOX, 12-lipoxygenase; 12/15-LOX, 12/15-lipoxygenase; 15-LOX, 15-lipoxygenase. Notes: General biosynthetic pathways and receptor assignments are supported by the cited review and primary biosynthesis literature. Ocular-surface evidence is summarized in the final column. * RvD6si is the nomenclature used in the original primary study; the same mediator is referred to as RvD6i in a subsequent review.

**Table 2:** Effects of Specialized Pro-Resolving Mediators on Conjunctival Goblet-Cell Function.

Mediator	Model System	Effect on [Ca^2+^]_i_	Effect on Glycoconjugate/ Mucin Secretion	Counter-Regulatory Activity	Key Signaling Pathway(s)	References
RvD1	Rat and human conjunctival goblet cells	Increase	Increase	Histamine: blocks calcium elevation, ERK1/2 activation in rat cells, and secretion. LTB4: blocks calcium elevation, not secretion.	EGFR transactivation -> AKT/ERK1/2; GPR32 in human cells; ALX/FPR2 in rat cells; βARK1/PKC for H1 counter-regulation.	[11,15,16]
RvE1	Rat conjunctival goblet cells; human calcium response reported	Increase	Increase	Blocks LTB4-induced calcium elevation and secretion.	ChemR23/ERV1 (CMKLR1) and BLT1; PLC/PLD/PLA2; EGFR-dependent ERK1/2 without EGFR transactivation; βARK1-mediated BLT1 counter-regulation.	[15,16,19]
LXA4	Human conjunctival goblet cells; rat and human histamine counter-regulation examined	Increase	Increase	Blocks histamine-induced calcium elevation and secretion; inhibits histamine-induced ERK1/2 activation in rat cells.	ALX/FPR2; ERK1/2; βARK1-mediated H1-receptor counter-regulation.	[13]
MaR1	Rat conjunctival goblet cells	Increase	Increase	Blocks histamine; blocks LTB4-induced calcium elevation.	BLT1 and ALX/FPR2; PLC/IP3, PKC, PLD, CaMKII, and ERK1/2.	[17,18]
MaR2	Rat conjunctival goblet cells	Increase	Increase	Blocks histamine; does not block LTB4-induced calcium elevation.	BLT1; cAMP/PKA, PLD, PLC-PKC, and PLA2. PLC-IP3 contributes to secretion but is not required for the calcium response.	[18]
Annexin A1	Rat conjunctival goblet cells	Increase	Increase	Not tested against histamine or LTB4 in the cited study.	ALX/FPR2; PLC/IP3, PKC, and CaMK for calcium; ERK1/2, PLA2, and PLD for secretion.	[12]

Abbreviations: AKT, protein kinase B; ALX/FPR2, lipoxin A4 receptor/formyl peptide receptor 2; βARK1, β-adrenergic receptor kinase 1; BLT1, leukotriene B4 receptor 1; Ca^2+^, calcium ion; CaMK, calcium/calmodulin-dependent protein kinase; CaMKII, calcium/calmodulin-dependent protein kinase II; cAMP, cyclic adenosine monophosphate; ChemR23/ERV1 (CMKLR1), chemerin chemokine-like receptor 1/E-series resolvin receptor 1 (chemokine-like receptor 1); EGFR, epidermal growth factor receptor; ERK1/2, extracellular signal-regulated kinase 1/2; GPR32, G-protein-coupled receptor 32; H1, histamine H1 receptor; IP3, inositol trisphosphate; LTB4, leukotriene B4; LXA4, lipoxin A4; MaR1, maresin 1; MaR2, maresin 2; PKC, protein kinase C; PKA, protein kinase A; PLA2, phospholipase A2; PLC, phospholipase C; PLD, phospholipase D; RvD1, resolvin D1; RvE1, resolvin E1; SPM, specialized pro-resolving mediator. Notes: Counter-regulatory activity refers only to the specific inflammatory agonists tested in the cited experiments. “Not tested” indicates that histamine- or LTB4-directed counter-regulation was not evaluated in the cited Annexin A1 study. Outcomes are from cultured rat and/or human conjunctival goblet-cell models unless otherwise stated.

## Data Availability

No new data were created or analyzed in this study. Data sharing is not applicable to this article.

## Abbreviations

AA, arachidonic acid; AKT, protein kinase B; ALX/FPR2, lipoxin A4 receptor/formyl peptide receptor 2; Alox15, arachidonate 15-lipoxygenase gene; AT, aspirin-triggered; BDNF, brain-derived neurotrophic factor; BCRP, breast cancer resistance protein; βARK1, β-adrenergic receptor kinase 1; BLT1, leukotriene B4 receptor 1; H1, histamine H1 receptor; FPR2, formyl peptide receptor 2; Ca^2+^, calcium ion; [Ca^2+^]_i_, intracellular calcium concentration; CaMK, calcium/calmodulin-dependent protein kinase; CaMKII, calcium/calmodulin-dependent protein kinase II; cAMP, cyclic adenosine monophosphate; CD4, cluster of differentiation 4; CD11b, cluster of differentiation 11b; ChemR23/ERV1, chemerin receptor 23/E-series resolvin receptor 1; CMKLR1, chemerin chemokine-like receptor 1; COX-2, cyclooxygenase-2; CYP450, cytochrome P450; CXCL1, C-X-C motif chemokine ligand 1; DED, dry eye disease; DHA, docosahexaenoic acid; EGF, epidermal growth factor; EGFR, epidermal growth factor receptor; EPA, eicosapentaenoic acid; ERK1/2, extracellular signal-regulated kinase 1/2; FLT4, fms-related receptor tyrosine kinase 4; GPCR, G-protein-coupled receptor; GPR32, G-protein-coupled receptor 32; HSV-1, herpes simplex virus 1; ICAM-1, intercellular adhesion molecule 1; IκBα, inhibitory kappa B alpha; IL, interleukin; IP3, inositol trisphosphate; LC-MS/MS, liquid chromatography-tandem mass spectrometry; LFA-1, lymphocyte function-associated antigen 1; LOX, lipoxygenase; LPS, lipopolysaccharide; LTB4, leukotriene B4; LXA4, lipoxin A4; LXB4, lipoxin B4; M1, type 1 macrophage; M2, type 2 macrophage; MaR1, maresin 1; MaR2, maresin 2; MCP-1, monocyte chemoattractant protein 1; miRNA, microRNA; MIP-2, macrophage inflammatory protein 2; MMP-9, matrix metalloproteinase 9; mRNA, messenger RNA; MRP2, multidrug resistance-associated protein 2; NADPH, nicotinamide adenine dinucleotide phosphate; NF-κB, nuclear factor kappa B; NGF, nerve growth factor; NPD1, neuroprotectin D1; OCT1, organic cation transporter 1; PD1, protectin D1; PEDF, pigment epithelium-derived factor; PKA, protein kinase A; PKC, protein kinase C; PLA2, phospholipase A2; PLC, phospholipase C; PLD, phospholipase D; PRK, photorefractive keratectomy; pSTAT1, phosphorylated signal transducer and activator of transcription 1; RvD1-RvD6, D-series resolvins 1–6; RvD6si, resolvin D6 stereoisomer; RvE1, resolvin E1; RvE2, resolvin E2; RX-10005, resolvin E1 methyl ester prodrug; RX-10045, resolvin E1 analog; RNA, ribonucleic acid; Sirt1, sirtuin 1; SPM, specialized pro-resolving mediator; Th1, T helper type 1; Th17, T helper type 17; TNF-α, tumor necrosis factor alpha; VEGF-A, vascular endothelial growth factor A; 15R-HETE, 15R-hydroxyeicosatetraenoic acid; 17S-HDHA, 17S-hydroxydocosahexaenoic acid; 17S-HpDHA, 17S-hydroperoxydocosahexaenoic acid; 18R-HEPE, 18R-hydroxyeicosapentaenoic acid; 5-LOX, 5-lipoxygenase; 12-LOX, 12-lipoxygenase; 12/15-LOX, 12/15-lipoxygenase; 15-LOX, 15-lipoxygenase.
